# Can large language models be trusted? Reliability and readability of responses to perinatal depression FAQs

**DOI:** 10.3389/fpubh.2026.1760872

**Published:** 2026-02-23

**Authors:** Jingyu Huang, Hua Yu, Junjian Chen, Xinyue Wang, Lizhi Huang, Junjie Wen, Hui Li

**Affiliations:** 1 Faculty of Health Sciences, University of Macau, Taipa, China; 2 Department of Obstetrics, Ruikang Hospital, Guangxi University of Chinese Medicine, Nanning, China; 3 Second Affiliated Hospital of Guangxi Medical University, Nanning, China; 4 GuangXi University of Chinese Medicine, Nanning, China; 5 Department of Medical Informatics, Harbin Medical University, Harbin, China; 6 Southwest Jiaotong University Hope College, Chengdu, China; 7 Department of Nursing, Ruikang Hospital Affiliated to Guangxi University of Chinese Medicine, Nanning, China

**Keywords:** generative artificial intelligence, health information quality, large language models, perinatal depression, postpartum depression, readability

## Abstract

**Objective:**

Large language models (LLMs), a core technology of generative artificial intelligence (AI), are increasingly used in health education and promotion. Although they may expand access to medical information, concerns remain regarding the reliability and readability of AI generated content for the public. This study evaluated the reliability and readability of answers generated by five LLMs to common questions about perinatal depression. The primary aims were to determine (1) the reliability of LLM responses to frequently asked questions about perinatal depression and (2) whether the readability of the generated content aligns with public health literacy levels.

**Methods:**

Twenty-seven frequently asked questions were derived from Google Trends and patient facing resources from the American College of Obstetricians and Gynecologists (ACOG). Each question was submitted to ChatGPT-5, Gemini-2.5, Microsoft Copilot, Grok4, and DeepSeek. Two obstetricians independently rated responses using five validated instruments (DISCERN, EQIP, JAMA, GQS, and HONCODE) and inter-rater agreement was quantified using the interclass correlation coefficient (ICC). Readability was assessed using six indices: ARI, GFI, CLI, OLWF, LWGLF, and FRF. Differences among models were analyzed using the Friedman test.

**Results:**

Inter rater agreement was high across 27 perinatal depression questions. ICC values ranged from 0.729 to 0.847. Significant between model differences emerged for DISCERN, EQIP, and HONCODE. All had *p* less than 0.001. No overall differences were found for JAMA and GQS. Grok4 scored highest on DISCERN at 60.33 ± 5.48. DeepSeek scored highest on EQIP at 53.04 ± 4.91. Copilot scored highest on HONCODE at 9.26 ± 1.85. These results highlight distinct strengths in quality constructs across instruments. Readability posed a common limitation. All models exceeded the NIH recommended sixth grade level on grade-based indices (for example, ARI ranged from 13.49 ± 2.92 to 15.81 ± 3.25). Similarly, OLWF scores fell well below the sixth-grade benchmark of 94 (ranging from 61.44 ± 6.80 to 72.96 ± 10.39, where higher scores denote easier reading). Most models produced empathetic and informative content. However, they fell short in fully addressing clinical safety standards.

**Conclusion:**

Most LLMs demonstrated moderate to high reliability when responding to perinatal depression questions, supporting their potential as supplementary sources of health information. However, readability levels above recommended benchmarks suggest that current outputs may remain challenging for individuals with lower health literacy. While LLMs improve information accessibility, further improvements in readability, source attribution, and ethical transparency are needed to maximize public benefit and support equitable health communication. Future work should focus on defining and standardizing safety behaviors in high-risk mental health contexts to enable reliable clinical deployment.

## 1 Introduction

Perinatal depression refers to depressive symptoms occurring during pregnancy or within 1 year postpartum, encompassing both antenatal and postpartum depression (1). This mood disorder not only undermines maternal mental health but also affects fetal and infant development (2). It may disrupt neural networks involved in emotion regulation and is associated with poorer offspring outcomes, including reduced attentional control, impaired emotion regulation, and altered social responsiveness (3). Globally, approximately 10%–20% of women experience perinatal depression (4). In low and middle-income countries, the overall prevalence is 24.7%, substantially higher than in high-income countries (5).

Among high-risk groups including racial/ethnic minorities, low-income populations, adolescent mothers, individuals with unintended pregnancies, and survivors of intimate partner violence the prevalence of perinatal depression is higher. A 2025 study reported that the prevalence of postpartum depression among adolescent mothers reached 40% and was even higher among minority and low-income adolescents (6). Unintended pregnancy is associated with an approximately 51%–59% increase in the relative risk of perinatal depression (7), and the prevalence among individuals exposed to intimate partner violence has been reported as 38.9% (8). Overall, these high-risk populations commonly exhibit prevalence rates in the range of approximately 30%–40%.

Access to treatment for perinatal depression remains insufficient worldwide, and minority groups and low-income women face substantial barriers to obtaining mental health services during pregnancy and the postpartum period (9–11). A systematic review highlighted the effectiveness of psychotherapy for postpartum depression but noted that, due to limited-service availability, cultural barriers, and disparities in resources, only a minority of patients can receive psychotherapy (12). Psychological, economic, and social factors often deter individuals from seeking professional help. Common psychological barriers include stigma, fear, and misconceptions about perinatal depression, all of which significantly reduce willingness to seek mental health support among perinatal women (13–15). Financial constraints, such as inadequate insurance coverage and the out-of-pocket cost of psychotherapy, also impede help seeking (16). In low and middle-income countries, the economic burden of perinatal depression is substantial, including direct treatment costs and productivity losses, and represents a major structural barrier to care (17). Social stigma and cultural attitudes, such as limited support from partners or family, fear of community judgment, and the stigmatization of postpartum mental health problems, further restrict access to care (13, 18, 19).

With the rapid development of artificial intelligence, obtaining health information through AI has become a simple and potentially effective alternative, and is increasingly adopted by the public (20). Generative artificial intelligence (GenAI) refers to a new generation of machine learning systems, such as large language models, that can generate novel, human like text rather than merely retrieving pre-existing information from a database. These models can synthesize complex medical information into conversational responses, making them increasingly popular for patient health education (21). As an emerging technology, GenAI has been reported to enhance the accessibility and dissemination of health information (22).

Generative AI (GenAI) has attracted substantial attention in recent years as a key branch of artificial intelligence in healthcare, with applications spanning diagnostic support, health education, image synthesis, and clinical decision making (23, 24). In perinatal mental health, GenAI has been explored for potential use in delivering personalized informational support and assisting with screening-oriented interactions aimed at identifying and addressing postpartum depression, which may improve the efficiency of psychosocial support (25). It can generate patient friendly health summaries in response to user queries, potentially supporting clinical communication and self-management (26). A systematic review noted that in perinatal mental health settings, where risk communication and information reliability are particularly critical, GenAI may improve accessibility to information and the comprehensibility of content, but its real world effectiveness and safety boundaries require more rigorous evaluation (27). In addition, GenAI chatbots may provide health education and conversational support, which could help improve access to information and health literacy; however, evidence for benefits on clinical outcomes, such as psychological recovery or sustained behavior change, remains limited and warrants further validation under appropriate governance and risk controls (28).

Despite the established potential benefits of GenAI, research on the quality and readability of AI generated responses to perinatal depression–related queries remain limited. One study evaluated AI models and search engines in the context of postpartum depression using a single quality metric and did not assess readability (29). Overall, evidence regarding the quality and readability of LLM responses to frequently asked questions about perinatal depression is still scarce. Given the public health burden of perinatal depression, the present study systematically compared the reliability and readability of responses generated by different models. We benchmarked five state of the art LLMs, ChatGPT-5, Gemini-2.5, Microsoft Copilot, Grok4, and DeepSeek, on 27 commonly searched perinatal depression queries derived from Google Trends and the American College of Obstetricians and Gynecologists (ACOG) website. This work provides foundational evidence to inform the use of AI-generated health information and to support future research on safety and effectiveness.

## 2 Materials and methods

Perinatal depression includes both antenatal and postpartum depression. We identified 27 publicly available questions related to perinatal depression using a two step approach. First, we queried Google Trends using the terms “prenatal depression” and “postpartum depression,” with the time range set from 2020 to May 2025 and the geographic scope set to worldwide, which yielded 25 candidate questions. Duplicate, irrelevant, or nonsensical entries were removed. Second, we searched the American College of Obstetricians and Gynecologists (ACOG) website for the sections on “Postpartum Depression” and “Depression During Pregnancy (30, 31).” After excluding items overlapping with the Google Trends results and screening the remaining items, we obtained a final set of 27 questions. The 27 unique questions (Table 1) were submitted verbatim to each LLM.

**Table 1:** Common public questions about perinatal depression (*n* = 27).

1. What is depression?
2. What is postpartum depression?
3. What is perinatal depression?
4. How common is depression during pregnancy?
5. What are the signs of depression during pregnancy?
6. How can untreated depression affect me during pregnancy?
7. How can untreated depression affect my fetus and newborn?
8. How can I get help for depression during pregnancy?
9. How is depression during pregnancy treated?
10. What is psychotherapy?
11. What are antidepressants?
12. What should I know about taking an antidepressant during pregnancy?
13. Can antidepressants pass to a baby through breast milk?
14. Can antidepressants cause side effects?
15. What other mental health conditions are common during pregnancy?
16. What is perinatal anxiety?
17. What is perinatal mental health?
18. What is prenatal depression?
19. What are perinatal depression symptoms?
20. What are the baby blues?
21. How long do the baby blues usually last?
22. When does postpartum depression occur?
23. What causes postpartum depression?
24. I think I have postpartum depression. What should I do?
25. How is postpartum depression treated?
26. What can be done to help prevent postpartum depression in women with a history of depression?
27. What support is available to help me cope with postpartum depression?

### 2.1 Experimental setup and model evaluation

All interactions were conducted in Macau, China, between November 1 and November 10, 2025. To approximate typical end user usage, all models were evaluated via their official web interfaces rather than *via* APIs. The evaluated products included ChatGPT using GPT-5, released on August 7, 2025; Gemini 2.5 Pro, released on June 17, 2025; DeepSeek (V3.1), released on August 21, 2025; Grok 4, released on July 9, 2025; and Microsoft Copilot. All products were commercially deployed closed source systems, so training data and model parameters were not accessible and generation settings such as temperature and top p remained at platform defaults. Each question was asked in a single turn without follow up prompts and submitted in a new chat session to minimize carry over effects. Outputs were captured verbatim via scripted logging with manual cross checking. We did not actively enable any explicit browsing or citation modes. However, in closed source web products the backend retrieval and routing behavior cannot be fully verified; Microsoft documentation indicates that Copilot Chat runs on GPT-5 by default and automatically routes prompts to the best performing models for each task, so the exact model and routing path used for any given response cannot be reliably identified or controlled, which may limit strict reproducibility.

### 2.2 Data handling and analysis

This study employed a double-blind design to ensure objective evaluation and minimize bias. The data from each model’s output were anonymized by an independent third party to eliminate any identifying information. The scoring process was carried out by two trained clinical evaluators who were blinded to the model type, prompt variations, search augmentation, generation parameters, and any metadata. Both evaluators, each with more than 10 years of clinical experience and based at tertiary (Grade A) hospitals in China, independently assessed the outputs. They had extensive experience in obstetrics and some research experience in perinatal maternal mental health. Prior to scoring, the evaluators received standardized training on the use of the assessment instruments. Any discrepancies in ratings were adjudicated by a third senior physician. Reliability was assessed using five validated tools; detailed scoring criteria are provided in Table 2.

**Table 2:** Scoring ranges and interpretation thresholds for reliability quality instruments used to evaluate LLM generated responses.

Tool	Score range	Excellent	Good	Fair	Poor
DISCERN	16–80	63–80	50–62	31–49	16–30
EQIP	0–100	76–100	51–75	26–50	0–25
JAMA	0–4	4	3	2	0–1
GQS	1–5	5	4	3	1–2
HONCODE	0–16	13–16	9–12	5–8	0–4

### 2.3 Evaluation criteria and statistical analysis

DISCERN is used to evaluate consumer health publications, particularly the quality of information on treatment choices (32). The instrument comprises 16 items grouped into three domains: reliability of the publication, quality of information on treatment options, and an overall rating. Each item is scored from 1 (lowest) to 5 (highest), yielding a total score ranging from 16 to 80.

The EQIP (Ensuring Quality Information for Patients) tool assesses the quality of online patient information. Developed by Moult et al. (33) in 2004, EQIP includes 20 items designed to evaluate the completeness, presentation, and usability of written patient information, and it has been widely used in health information quality assessment studies (34, 35). Each item is rated using a binary yes/no criterion (“yes” = 5 points; “no” = 0 points). The overall score is calculated as the proportion of “yes” responses multiplied by 100, resulting in a total score ranging from 0 to 100.

The JAMA benchmarks, proposed by the *J*ournal of the American Medical Association in 1997, provide a simple framework for assessing the quality of online health information sources (36). They evaluate four transparency components: authorship, attribution, disclosure, and currency. Each criterion is scored as 1 if present and 0 if absent, yielding a total score from 0 to 4, with higher scores indicating better information quality.

GQS (The Global Quality Score), developed by Bernard and colleagues in 2007, is a subjective overall rating tool commonly used to assess the quality of online health information, particularly in internet-based health promotion research (37). It provides a rapid global judgment of medical accuracy, completeness, and educational usefulness. GQS is typically rated on a 5-point Likert scale, where 1 indicates very poor/misleading information and 5 indicates excellent/highly useful information.

HONCODE is maintained by the Health on the Net Foundation (founded in 1995) and is one of the oldest and most widely recognized certification standards for online medical and health information. It assesses compliance with eight ethical and transparency principles. Each principle is scored as 0, 1, or 2 (0 = not compliant; 1 = partially compliant; 2 = fully compliant), producing a total score ranging from 0 to 16, with higher scores indicating greater reliability and transparency (38).

Readability was evaluated using six indices, each reflecting a different aspect of textual complexity. The Automated Readability Index (ARI) estimates the grade level required to comprehend a text based on sentence length and word length. The Gunning Fog Index (GFI) reflects reading difficulty using average sentence length and the proportion of complex words. The Coleman–Liau Index (CLI) estimates grade level based on characters per word and sentences per text. The Original Linsear Write Formula (OLWF) captures the balance between word length and sentence length on a 0–100 scale. The Linsear Write Grade Level Formula (LWGLF) reflects difficulty based on the proportion of simple versus complex words. The FORCAST Readability Formula (FRF) evaluates readability based on word complexity and sentence structure.

The National Institutes of Health (NIH) recommends that public health information be written at approximately a sixth to seventh grade reading level (39).therefore, we adopted the sixth-grade level as our benchmark. For grade-based indices (ARI, GFI, CLI, LWGLF, and FRF), the output approximates U.S. School grade levels, with higher scores indicating more difficult text. Accordingly, a target value of 6 was used, and scores closer to 6 indicate better alignment with recommended public readability levels. For OLWF, higher scores indicate easier reading; based on the published correspondence between OLWF values and sixth-grade materials, we used OLWF = 94 as the sixth-grade benchmark. Using these benchmarks, we compared model outputs against the reference values in the Results: grade-based indices substantially above 6 indicate higher reading burden, whereas OLWF values substantially below 94 indicate insufficient readability.

Means and standard deviations were used as descriptive statistics to summarize reliability and readability scores. Given the repeated measures design, we used the Friedman test to assess overall differences among models, followed by paired Wilcoxon signed rank tests with Bonferroni adjustment for *post hoc* pairwise comparisons. Effect sizes (*r*) were calculated to quantify the magnitude of pairwise differences. Inter rater agreement between the two physicians for reliability ratings was assessed using the interclass correlation coefficient (ICC). Data analyses were performed in R (version 4.5.2).

## 3 Results

### 3.1 Reliability analysis

**Figure 1:**
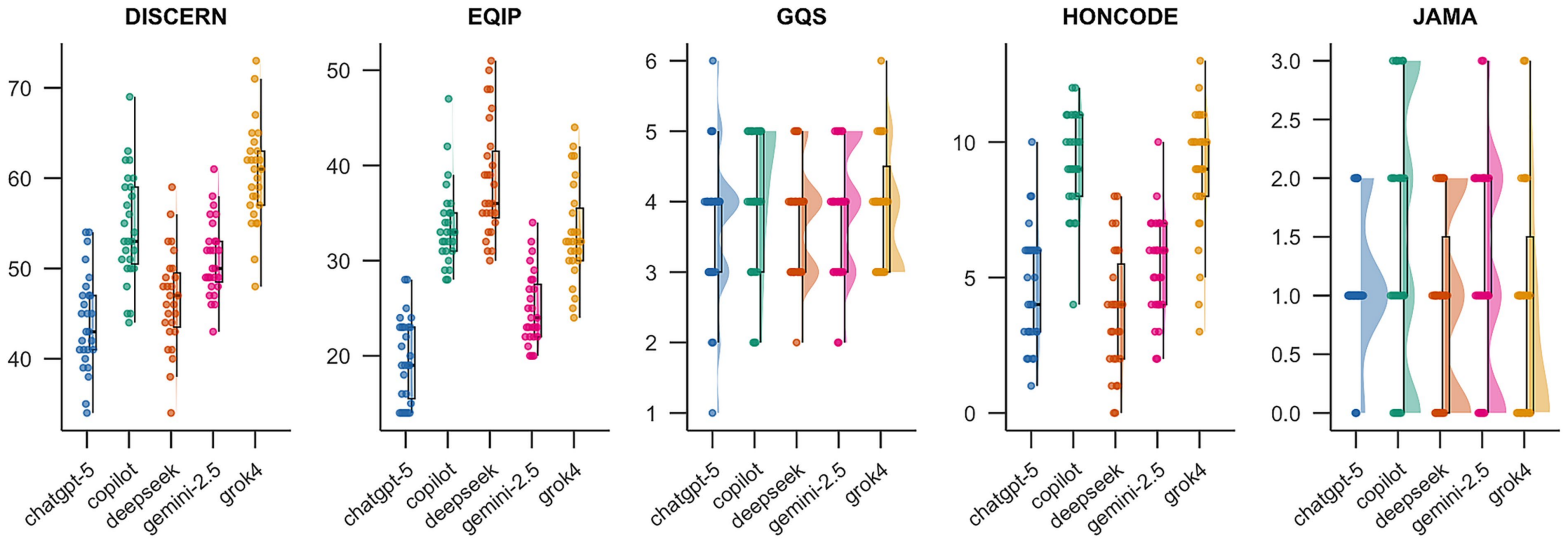
Distribution of reliability quality scores across five large language models (*n* = 27 questions). Scores were evaluated using DISCERN, EQIP, JAMA, GQS, and HONCODE. Higher scores indicate better information quality/credibility across instruments.

Reliability was assessed using five instruments: DISCERN, EQIP, JAMA, GQS, and HONCODE. Inter rater agreement between the two obstetricians was high across instruments, with ICC values of 0.787 for DISCERN, 0.847 for GQS, 0.797 for JAMA, 0.729 for EQIP, and 0.777 for HONCODE. Figure 1 summarizes model scores, and Table 3 reports overall between model differences using Friedman tests. Significant differences were observed for DISCERN, EQIP, and HONCODE (all *p* < 0.001), whereas overall differences were not significant for JAMA and GQS.

**Table 3:** Reliability scores (Mean ± SD) of large language models and overall comparisons using Friedman tests.

Model	DISCERN	EQIP	JAMA	GQS	HONCODE
ChatGPT-5	44.07 ± 5.31	50.11 ± 4.11	1.07 ± 0.55	3.67 ± 1.04	4.59 ± 2.22
Copilot	54.48 ± 6.06	52.41 ± 4.48	1.48 ± 1.09	3.96 ± 1.02	9.26 ± 1.85
DeepSeek	46.63 ± 5.47	53.04 ± 4.91	0.96 ± 0.76	3.70 ± 0.78	3.70 ± 2.32
Gemini-2.5	51.11 ± 4.17	47.81 ± 3.63	1.37 ± 1.08	3.78 ± 0.89	5.41 ± 1.87
Grok4	60.33 ± 5.48	52.18 ± 6.43	0.93 ± 0.96	4.00 ± 0.83	8.89 ± 2.36
Friedman *χ^2^* (df)	70.79 (4)	19.08 (4)	4.16 (4)	4.07 (4)	60.38 (4)
*p*	<0.001	<0.001	0.385	0.396	<0.001

For DISCERN, which reflects the completeness and quality of information about treatment options, models differed significantly in performance. Grok4 and Copilot achieved the highest scores, suggesting that their responses were more clearly structured and comprehensive, particularly in describing treatment options as well as potential risks and benefits. ChatGPT-5 scored the lowest on this dimension; overall scores fell in the good to fair range, implying room for improvement in content depth and information organization.

For EQIP, DeepSeek performed best in clarity of expression, formatting standardization, and logical flow, followed by Copilot and Grok4. Gemini-2.5 scored the lowest, reflecting limitations in readability, information structure, and explanatory detail. Overall, except for Gemini-2.5 and ChatGPT-5, most models were within the good range, indicating generally acceptable clarity but with opportunities for further optimization.

For JAMA, which emphasize authority and transparency, Copilot and Gemini-2.5 performed relatively better, particularly with respect to authorship identification, source attribution, and disclosure statements, aligning more closely with standards commonly expected in medical publishing. Grok4 scored the lowest on this dimension, which may reflect more limited or conservative practices in providing explicit attribution and disclosures within its responses.

For GQS, Grok4 and Copilot scored slightly higher than other models in overall perceived information quality, including usefulness and user perceived value. ChatGPT-5 was relatively weaker, suggesting that it may require improvement in conveying key clinical details effectively. Most models were rated in the fair range, and mean differences were modest, indicating that the generated health information was generally adequate but not consistently high quality.

For HONCODE, which evaluates ethical and transparency principles of health information, Copilot and Grok4 performed best, scoring significantly higher than other models, suggesting better alignment with expectations related to transparency, accountability, and information governance. Gemini-2.5 and ChatGPT-5 were slightly above average, whereas DeepSeek scored the lowest, indicating that ethical and transparency aspects may warrant further strengthening for some models.

Based on the Friedman results shown in Table 3 and the effect size analysis in Supplementary document, significant between model differences were observed in overall quality assessments, including DISCERN (*χ^2^* = 70.79, *p* < 0.001), EQIP (*χ^2^* = 19.08, *p* < 0.001), and HONCODE (*χ^2^* = 60.38, *p* < 0.001). These findings indicate that the evaluated models differed in their performance on quality constructs relevant to complex medical information.

*Post hoc* pairwise comparisons further showed that the magnitude of these differences varied across instruments. For DISCERN and HONCODE, several comparisons yielded large effect sizes (*r* > 0.6), particularly for model pairs involving Grok4 and Copilot, suggesting substantial differences in reliability related performance. For EQIP, effect sizes spanned large, moderate, and small ranges, indicating that this instrument provided meaningful discrimination among models. In contrast, no statistically significant overall differences were detected for the simpler, more subjective scales such as GQS (*p* = 0.396) and JAMA (*p* = 0.385). Consistent with this, *post hoc* analyses generally showed small or negligible effect sizes, suggesting that model performance was broadly comparable when evaluated using these basic quality benchmarks.

Table 4 presents Bonferroni corrected post hoc pairwise comparisons of reliability scores among the five large language models. Significant pairwise differences were primarily observed for DISCERN, EQIP, and HONCODE, whereas few significant differences were detected for JAMA and GQS. Notably, DeepSeek and ChatGPT-5 showed no significant differences across most reliability metrics, while Grok4 differed significantly from several other models on multiple measures.

**Table 4:** Post hoc pairwise comparisons of reliability scores among five LLMs using paired Wilcoxon signed rank tests (Bonferroni-adjusted *p*-values).

Comparison (model A *vs.* model B)	DISCERN	EQIP	JAMA	GQS	HONCODE
DeepSeek—Copilot	<0.01	1	0.81	1	<0.01
DeepSeek—ChatGPT-5	0.20	0.33	1	1	1
DeepSeek—Gemini-2.5	0.04	<0.01	1	1	0.08
DeepSeek—Grok4	<0.01	1	1	1	<0.01
Copilot—ChatGPT-5	<0.01	0.93	0.74	1	<0.01
Copilot—Gemini-2.5	0.07	<0.01	1	1	<0.01
Copilot—Grok4	<0.01	1	0.75	1	1
ChatGPT-5—Gemini-2.5	<0.01	0.45	1	1	1
ChatGPT-5—Grok4	<0.01	1	1	1	<0.01
Gemini-2.5—Grok4	<0.01	<0.01	0.99	1	<0.01

Values are Bonferroni-adjusted *p*-values (*p*_adj). Statistical significance was set at *p*_adj < 0.05.

### 3.2 Readability analysis

**Figure 2:**
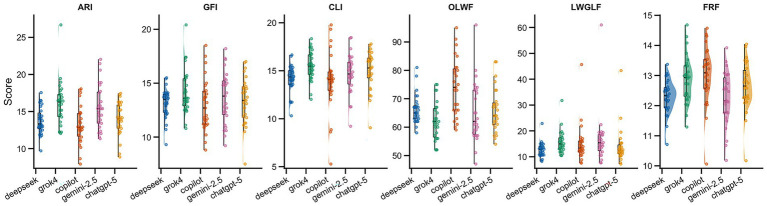
Distribution of readability metrics across five large language models with sixth-grade benchmarks (*n* = 27 questions). Indices include ARI, GFI, CLI, OLWF, LWGLF, and FRF. Higher scores indicate greater reading difficulty for ARI/GFI/CLI/LWGLF/FRF, whereas higher OLWF indicates better readability. Sixth-grade benchmark values are shown as reference lines.

Across the five AI models and six readability indices, detailed scores and Friedman test results are presented in Table 5 and Figure 2. None of the models achieved the recommended sixth-grade benchmark on ARI, GFI, CLI, OLWF, LWGLF, or FRF. Unlike the grade level indices, higher OLWF scores indicate easier readability; therefore, OLWF values substantially below the sixth-grade benchmark reflect increased reading difficulty. Overall, this indicates that text generated by all models exceeded the complexity expected for sixth grade reading level.

**Table 5:** Readability scores (Mean ± SD) of LLM generated responses across six indices, including a sixth-grade benchmark and overall comparisons using the Friedman test.

Model	ARI	GFI	CLI	OLWF	LWGLF	FRF
ChatGPT-5	13.98 ± 2.39	13.25 ± 2.39	14.88 ± 2.18	65.04 ± 7.42	13.47 ± 7.64	12.65 ± 0.94
Copilot	13.49 ± 2.92	13.00 ± 2.79	13.77 ± 3.39	72.96 ± 10.39	14.10 ± 7.78	12.83 ± 1.12
DeepSeek	13.88 ± 2.08	12.94 ± 1.63	13.91 ± 1.65	66.63 ± 5.77	12.74 ± 3.39	12.19 ± 0.60
Gemini-2.5	15.12 ± 2.90	13.07 ± 2.35	13.95 ± 2.20	64.96 ± 10.07	15.74 ± 10.72	12.21 ± 0.95
Grok4	15.81 ± 3.25	13.89 ± 2.39	15.37 ± 1.64	61.44 ± 6.80	15.44 ± 4.81	12.79 ± 0.76
6th grade level score	6	6	6	94	6	6
*p*	<0.001	0.064	<0.001	<0.001	0.003	<0.001
Friedman *χ*^2^ (*df*)	34.90 (4)	8.88 (4)	26.22 (4)	33.74 (4)	15.92 (4)	21.62 (4)

Grok4 and Gemini-2.5 obtained the highest scores on multiple indices, suggesting that they produced the most complex text and thus imposed the highest reading burden. Copilot and DeepSeek had slightly lower scores, but remained well above recommended levels, indicating marginally easier text that may nonetheless be unsuitable for individuals with lower health literacy. ChatGPT-5 was closer to the overall average on some indices, yet its outputs still tended toward higher complexity, particularly on CLI and ARI. Collectively, these findings suggest that model generated responses were generally complex and associated with a relatively high readability threshold.

The Friedman test results indicated statistically significant between model differences for ARI, CLI, OLWF, LWGLF, and FRF (all *p* < 0.01), whereas GFI did not reach statistical significance (*p* = 0.064). The non-significant GFI result likely reflects a shared characteristic across models: when responding to perinatal depression–related queries, all models inevitably used a substantial number of multisyllabic medical terms (e.g., “postpartum,” “antidepressant”), resulting in broadly similar levels of complex vocabulary. In contrast, indices more directly related to reading difficulty, such as ARI, CLI, and FRF, showed distinguishable differences across models, suggesting variability in linguistic complexity and reading burden.

By comparison, differences were smaller for indices that emphasize sentence structure and word combination features, such as GFI and LWGLF, indicating that models were broadly similar in average sentence length and in their overall control of complex word proportions. Effect size analyses further suggested that the magnitude of these differences was generally small to moderate, implying limited practical separation in readability across models. *Post hoc* pairwise comparisons showed that differences varied by readability index and did not yield a consistent pattern of superiority across all measures.

## 4 Discussion

The use of generative AI in mental health is expanding, with emerging applications in risk identification and early assessment of psychiatric disorders, personalized intervention and treatment support, and conversational emotional support. Prior research has shown that access to healthcare services among individuals with perinatal depression remains limited, suggesting a public need for AI assisted responses to perinatal depression–related questions. Therefore, the reliability and readability of AI generated information are critical for determining whether such content can meaningfully support the public. In this study, we evaluated the reliability and readability of information generated by five widely used AI models to characterize their performance on reliability related outcomes.

### 4.1 Safety related response characteristics of LLMs

There were substantial differences across large language models in how they handled safety critical perinatal mental health scenarios, particularly with respect to clinical appropriateness, risk communication, and referral to professional care. Although all models included general disclaimers that they cannot replace clinicians, they varied markedly in the depth of their clinical framing and in how closely they aligned with established care pathways.

Copilot and Grok4 provided the most comprehensive responses and were most consistent with clinical practice. Both models repeatedly emphasized that treatment decisions during pregnancy require individualized risk–benefit assessment under joint supervision by obstetric and psychiatric clinicians. Their responses explicitly acknowledged the risks of untreated perinatal depression for both the mother and the fetus, which is consistent with consensus guidance, while appropriately avoiding directive or prescriptive medical advice. ChatGPT consistently emphasized that it cannot replace a clinician and, in the context of postpartum depression, demonstrated strong safety awareness by encouraging timely professional evaluation, highlighting warning signs of self-harm, and directing users to crisis resources when indicated.

Grok4 performed particularly well in escalation behaviors, proactively providing region specific crisis hotlines and explicitly encouraging urgent care when severe symptoms were mentioned. It also attempted to identify nearby counseling or medical services by providing local addresses and phone numbers. However, Grok4’s responses were more variable in structure and sometimes prioritized extensive resource lists over a systematic clinical explanation, which may pose comprehension challenges for users with lower health literacy.

Gemini-2.5 generally recognized perinatal depression as a medical condition and encouraged help seeking; however, its responses were less consistent across similar prompts. Variation in emphasis across related questions suggests weaker internal consistency, and recommendations for crisis resources were less consistently embedded within an explicit clinical risk framework. Such inconsistency may limit the reliability of model outputs in high-risk mental health contexts, where stable and consistent safety signaling is essential.

DeepSeek’s responses were generally supportive and non-harmful, but they often remained at a broad educational level. Compared with other models, it less frequently provided explicit guidance on when urgent or emergency care is warranted, and its referral pathways were less specific. While this level of response may be acceptable for general psych education, it offers more limited support for risk recognition, stratification, and action planning when risk is elevated, or safety signals are ambiguous.

### 4.2 Reliability of information

Based on our results, Grok4 achieved the highest score under the DISCERN framework. As Grok4 is a relatively new model, there is currently limited published evidence available for direct comparison of DISCERN scores. The scoring profile of Grok4 suggests that its strengths are not primarily reflected in presentation style or formal citation conventions, but rather in its safety-oriented responses in high-risk scenarios. This characteristic may carry potential clinical value in perinatal mental health contexts, while also indicating room for improvement in the standardized presentation expected for public facing health education materials. In this sense, its outputs appear more aligned with a “clinical communication” style than an “educational material” style.

Among the remaining models, Copilot achieved the highest DISCERN score, outperforming Gemini-2.5 and DeepSeek, which is consistent with findings from two prior studies (40, 41). Copilot frequently emphasizes source attribution and often references academic journals and official guidelines; its strong performance on the JAMA further suggests that it strikes a balance between quality, breadth, and prioritization of key information. Although Gemini-2.5 obtained a relatively high JAMA score, it performed less well on DISCERN. This discrepancy may reflect the fact that DISCERN evaluates not only accuracy but also the structured, user oriented, and comprehensive presentation of information, including discussion of risks and alternative options. Thus, while Gemini-2.5 may perform well on transparency related criteria captured by JAMA, limitations in organization, user centered framing, or comprehensiveness may contribute to a lower DISCERN score. DeepSeek performed particularly well on EQIP, a pattern also reported in prior work. Its outputs resemble a “health education/health literacy” mode rather than a “clinical decision support” mode, which may be well suited for public health education.

Joint analysis of DISCERN and HONCODE showed a positive association between the two scores, consistent with previous findings (42). High performing models such as Grok4 and Copilot ranked higher on both instruments, whereas lower-performing models such as ChatGPT-5 tended to score lower on both. This suggests some overlap between information quality and adherence to established standards in consumer health contexts: higher-quality information is often more likely to align with transparency and accountability principles. A high DISCERN score indicates that the model’s content is reliable, well organized, and comprehensive, while a high HONCODE score indicates stronger alignment with ethical and transparency standards, such as attribution and authority. Together, these properties may increase public confidence and reduce exposure to misleading or incorrect information. If AI systems can further improve transparency and authority signaling, they may help bridge the gap between public understanding and professional medical knowledge—an especially important goal in an environment of information overload, uneven online content quality, lower health literacy in parts of the population, and persistent information asymmetry.

### 4.3 Readability of information

Readability is critical to determining whether health information can be effectively communicated. The NIH explicitly recommends that public health materials be written at approximately a sixth to seventh grade reading level to ensure that the public, particularly individuals with lower health literacy, can understand medical content (43). In our study, readability scores for all AI generated responses exceeded the sixth-grade level, with most approaching a high school reading level. This pattern is inconsistent with the NIH recommended benchmark for public facing health information. When the readability of health information exceeds the public’s reading ability, several problems may arise. First, comprehension barriers may occur information may be available but not truly accessible, and readers may struggle with vocabulary, sentence structure, or logical organization. Even well-educated readers may misinterpret specialized medical terminology, treatment recommendations, or risk explanations, leading to misunderstanding or disregard of key information. Second, trust may be undermined: overly technical content can appear less trustworthy, and highly complex text may create psychological distance or trigger skepticism, especially when source attribution, update dates, or author credentials are unclear, thereby reducing acceptability and communication effectiveness (44). Third, health behaviors may be impeded: information may be encountered but not translated into action. Poor readability reduces usability, making it difficult for lay readers to extract actionable points, which can weaken adherence and limit behavior change. This concern is particularly salient for perinatal women, who may experience substantial psychological stress; when health information is obscure or difficult to understand, uncertainty and anxiety may increase. In contrast, clear and plain language information can be reassuring. Low readability may also lead pregnant or postpartum individuals to misinterpret advice regarding medications, diet, or psychological interventions, potentially resulting in poor adherence or avoidable risk. By improving readability, AI based tools may help reduce such misunderstandings. Moreover, high quality, readable information may enable healthcare professionals to guide women toward reliable resources more efficiently, potentially reducing clinical workload. Therefore, to maximize the public health value of AI generated content, future efforts should prioritize improving readability without compromising information quality, ensuring that medical information is both accurate and understandable.

### 4.4 Strengths

This study examined the reliability and readability of AI generated responses to perinatal depression related questions. Although numerous information quality assessments have been conducted in other medical domains, evidence in perinatal mental health remains limited. We evaluated five widely used, up to date AI models using well established instruments for reliability and readability, providing practical evidence to inform public facing consultation and the use of AI generated health information in this context.

### 4.5 Limitations

This study has several limitations. First, we interacted with commercially deployed models through their official web interfaces; as a result, generation parameters such as temperature and top p were governed by platform defaults, and the generation process could not be fully standardized. Second, closed-source web products such as Microsoft Copilot may employ platform-level model selection and dynamic routing, and specific underlying model versions and routing behaviors are not fully disclosed. Therefore, in a web-based setting, researchers cannot reliably identify or control the exact model and routing pathway used for each response, which may affect strict comparability and reproducibility across models. In addition, LLM outputs may vary over time due to silent platform updates, policy changes, or routing adjustments; thus, our findings reflect performance within a specific time window. Third, this evaluation was conducted only in English and used a limited FAQ set derived from Google Trends and ACOG resources, which may not capture different cultural contexts, varying levels of health literacy, or higher risk clinical scenarios. Finally, while we focused on reliability and readability, we did not systematically quantify safety critical elements in perinatal mental health contexts, such as crisis resource guidance, risk communication, or referral recommendations. Future studies should extend this work across multiple languages and time points, under more controllable generation settings, and incorporate explicit safety metrics to further validate and generalize our findings.

## 5 Conclusion

This study indicates that most AI models performed reasonably well when answering questions about perinatal depression. However, even the strongest performing models exhibited notable weaknesses; for example, Grok4 would benefit from improved performance on the JAMA, and Copilot would benefit from improvement on EQIP. Overall, readability across models was suboptimal. To enable the public to use AI more effectively and conveniently to obtain health information, further technical advances are needed to improve the readability of generated content. In addition, models should provide clearer source attribution by citing authoritative journals and official guidelines and by standardizing how sources and evidence are presented. With continued technical development, AI systems may better support public access to reliable health information.

Overall, although the evaluated LLMs were generally capable of generating empathetic and information rich content related to perinatal mental health, only some models consistently met key clinical safety expectations. These include appropriate referral guidance, discouraging self-directed medical decision making, and maintaining alignment with established care pathways. Such variability underscores the need for cautious deployment of generative AI in maternal mental health and highlights the importance of evaluating specific models rather than assuming equivalence across systems. Future research should focus on defining and standardizing safety behaviors, particularly in high-risk mental health scenarios, to ensure that AI generated health information supports informed decisions without inadvertently undermining safe clinical care.

## Data Availability

The raw data supporting the conclusions of this article will be made available by the authors, without undue reservation.
