# Correction: Can large language models be trusted? Reliability and readability of responses to perinatal depression FAQs

**DOI:** 10.3389/fpubh.2026.1822049

**Published:** 2026-03-24

**Authors:** Jingyu Huang, Hua Yu, Junjian Chen, Xinyue Wang, Lizhi Huang, Junjie Wen, Hui Li

**Affiliations:** 1 Faculty of Health Sciences, University of Macau, Taipa, China; 2 Department of Obstetrics, Ruikang Hospital, Guangxi University of Chinese Medicine, Nanning, China; 3 Second Affiliated Hospital of Guangxi Medical University, Nanning, China; 4 GuangXi University of Chinese Medicine, Nanning, China; 5 Department of Medical Informatics, Harbin Medical University, Harbin, China; 6 Southwest Jiaotong University Hope College, Chengdu, China; 7 Department of Nursing, Ruikang Hospital Affiliated to Guangxi University of Chinese Medicine, Nanning, China

**Keywords:** generative artificial intelligence, health information quality, large language models, perinatal depression, postpartum depression, readability

Affiliation for the author “Hua Yu” was erroneously given as “Department of Nursing, Ruikang Hospital Affiliated to Guangxi University of Chinese Medicine, Nanning, China” instead of “Department of Obstetrics, Ruikang Hospital, Guangxi University of Chinese Medicine, Nanning, China”.

The original version of this article has been updated.

